# Supplementary material for: UHRF1 Is a Sensor for DNA Interstrand Crosslinks and Recruits FANCD2 to Initiate the Fanconi Anemia Pathway
Source: Cell Rep. 2015 Mar 19;10(12):1947–56. doi: 10.1016/j.celrep.2015.02.053 (PMC4386029; doi:10.1016/j.celrep.2015.02.053)
Supplement: Document S1. Figure S1 [file mmc1.pdf]

Cell Reports

Supplemental Information

UHRF1 Is a Sensor for DNA Interstrand Crosslinks and Recruits FANCD2 to Initiate the Fanconi Anemia Pathway

Chih-Chao Liang, Bao Zhan, Yasunaga Yoshikawa, Wilhelm Haas, Steven P. Gygi, and Martin A. Cohn

Figure S1

A

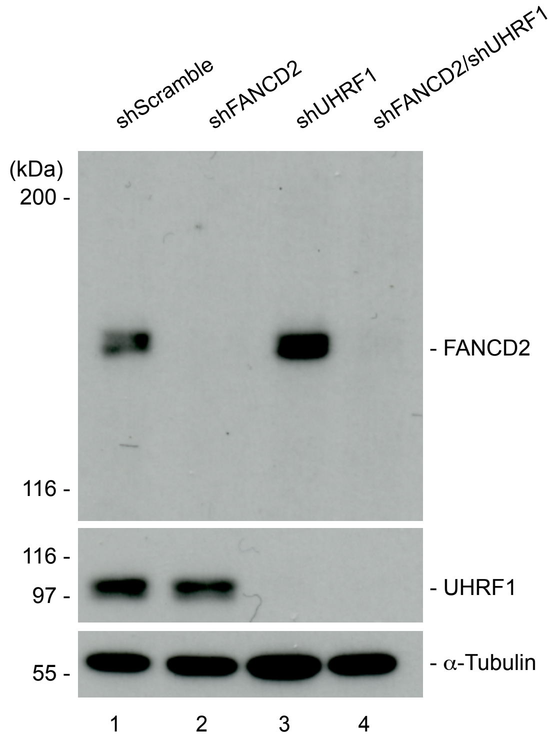

shScramble
shFANCD2
shUHRF1
shFANCD2/shUHRF1
(kDa)
200 -
116 -
- FANCD2
116 -
97 -
- UHRF1
55 -
- α-Tubulin
1
2
3
4

B

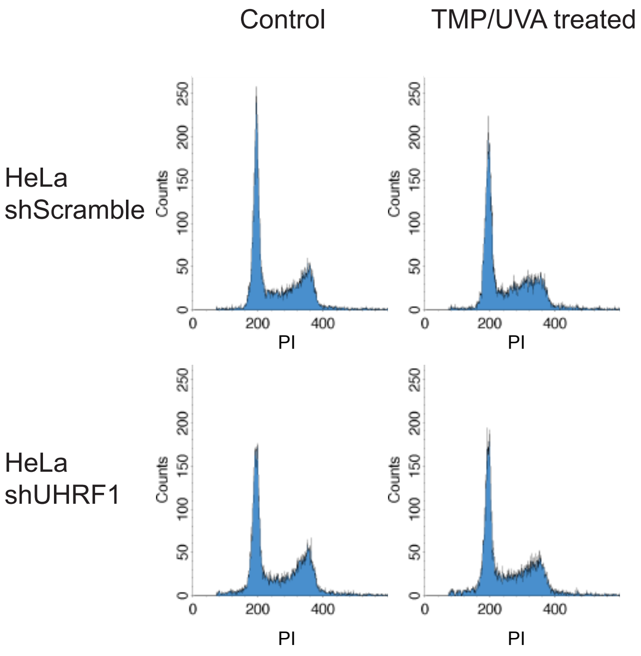

Control
TMP/UVA treated
HeLa shScramble
HeLa shUHRF1
Counts
0
50
100
150
200
250
0
200
400
PI

Supplementary figure legend

Figure S1, related to Figures 3, 4 and 5

A) Western blot analysis confirms the knock down level of UHRF1 and FANCD2 in the single and double knock down cells used in Figure 3G. B) HeLa.shScramble and HeLa.shUHRF1 were untreated or treated with TMP/UVA. Cells were harvested after 3 hours, stained with propidium iodide, and analyzed by FACS analysis.
