# Supplementary material for: UHRF1 Is a Sensor for DNA Interstrand Crosslinks and Recruits FANCD2 to Initiate the Fanconi Anemia Pathway
Source: Cell Rep. 2015 Mar 19;10(12):1947–56. doi: 10.1016/j.celrep.2015.02.053 (PMC4386029; doi:10.1016/j.celrep.2015.02.053)
Supplement: Document S2. Article plus Supplemental Information [file mmc2.pdf]

Cell Reports

Report

UHRF1 Is a Sensor for DNA Interstrand Crosslinks and Recruits FANCD2 to Initiate the Fanconi Anemia Pathway

Graphical Abstract

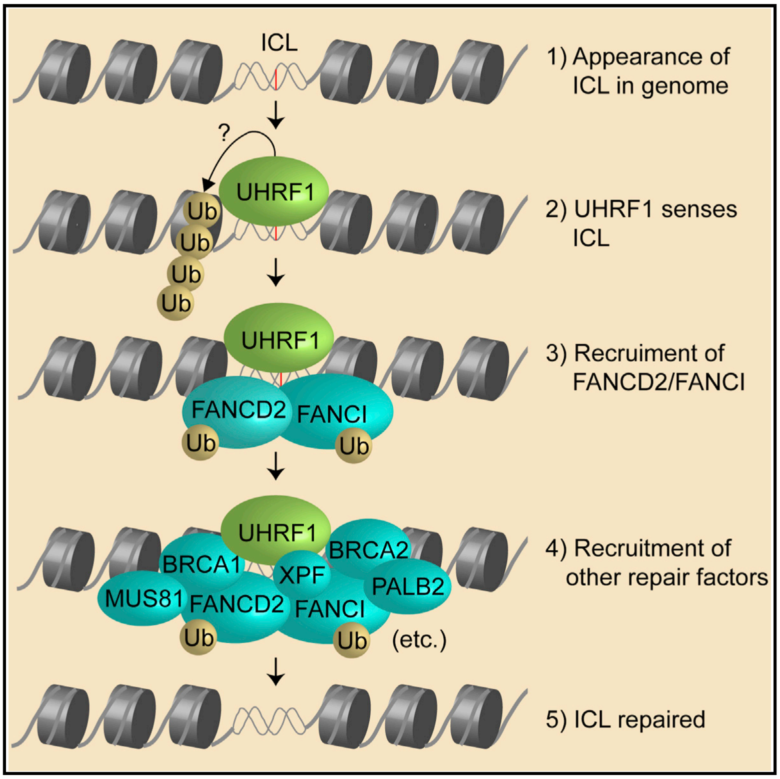

Highlights

- UHRF1 is a sensor for DNA interstrand crosslinks (ICLs)
- UHRF1 is recruited to ICLs within seconds of their appearance in the genome
- Recruitment of UHRF1 is required for proper recruitment of FANCD2 to ICLs
- UHRF1 is an integral part of the Fanconi anemia DNA repair pathway

Authors

Chih-Chao Liang, Bao Zhan, ..., Steven P. Gygi, Martin A. Cohn

Correspondence

martin.cohn@bioch.ox.ac.uk

In Brief

The Fanconi anemia pathway responds to DNA interstrand crosslinks (ICLs). Here, Liang et al. report the identification of UHRF1 as a protein that senses ICLs and show that its recruitment is necessary for the recruitment of FANCD2 to ICLs.

Liang et al., 2015, Cell Reports *10*, 1947–1956
March 31, 2015 ©2015 The Authors
http://dx.doi.org/10.1016/j.celrep.2015.02.053

OPEN ACCESS
CellPress

UHRF1 Is a Sensor for DNA Interstrand Crosslinks and Recruits FANCD2 to Initiate the Fanconi Anemia Pathway

Chih-Chao Liang,[1,3] Bao Zhan,[1,3] Yasunaga Yoshikawa,[1,3] Wilhelm Haas,[2] Steven P. Gygi,[2] and Martin A. Cohn[1,*]
[1]Department of Biochemistry, University of Oxford, Oxford OX1 3QU, UK
[2]Department of Cell Biology, Harvard Medical School, Boston, MA 01125, USA
[3]Co-first author
*Correspondence: martin.cohn@bioch.ox.ac.uk
http://dx.doi.org/10.1016/j.celrep.2015.02.053
This is an open access article under the CC BY license (http://creativecommons.org/licenses/by/4.0/).

SUMMARY

The Fanconi anemia (FA) pathway is critical for the cellular response to toxic DNA interstrand crosslinks (ICLs). Using a biochemical purification strategy, we identified UHRF1 as a protein that specifically interacts with ICLs in vitro and in vivo. Reduction of cellular levels of UHRF1 by RNAi attenuates the FA pathway and sensitizes cells to mitomycin C. Knockdown cells display a drastic reduction in FANCD2 foci formation. Using live-cell imaging, we observe that UHRF1 is rapidly recruited to chromatin in response to DNA crosslinking agents and that this recruitment both precedes and is required for the recruitment of FANCD2 to ICLs. Based on these results, we describe a mechanism of ICL sensing and propose that UHRF1 is a critical factor that binds to ICLs. In turn, this binding is necessary for the subsequent recruitment of FANCD2, which allows the DNA repair process to initiate.

INTRODUCTION

Interstrand crosslinks (ICLs) of the Watson-Crick DNA helix are extremely toxic to the genome. Consequently, humans have evolved effective mechanisms to respond to and repair such DNA damage. One such mechanism is the Fanconi anemia (FA) DNA repair pathway, which when deregulated causes the FA disease. FA is a recessive cancer predisposition and developmental syndrome that is characterized by hypersensitivity to DNA interstrand crosslinking agents (Cohn and D'Andrea, 2008). Proteins mutated in 17 FA complementation groups work together to ensure the repair of ICLs, a process that likely involves nucleotide excision repair, translesion synthesis, and homologous recombination. Central to the pathway are the FANCD2 and FANCI proteins. Upon DNA damage, these proteins are monoubiquitinated by the FA core E3 ubiquitin ligase complex, which is comprised of eight other FA proteins. After ubiquitination, FANCD2, FANCI and the remaining seven FA proteins are recruited to the ICLs (Ciccia et al., 2007). It is clear that the repair process can be initiated and performed both in a replication dependent (Knipscheer et al., 2009; Räschle et al., 2008) and replication independent process (Muniandy et al., 2009; Vasquez, 2010). We set out to identify proteins that are able to specifically recognize ICLs and thus might serve as sensors for such adducts. Here, we report the identification of UHRF1 as a protein that specifically recognizes ICLs in vitro and is required for effective repair of ICLs in vivo.

RESULTS

Purification of UHRF1

To purify a potential sensor protein for ICLs we developed a biochemical purification scheme (Figure 1A). The strategy is based on the assumption that an ICL forces the DNA to adopt a structure sufficiently different from that of a Watson-Crick double helix to provide enhanced binding properties for such a protein. We designed a 5′-biotinylated double-stranded DNA molecule containing a unique central TA sequence (Figure 1B). Incubation of the DNA with the psoralen derivative 4,5′,8-trimethylpsoralen (TMP), which intercalates specifically at the TA sequence, followed by exposure to ultraviolet A (UVA) irradiation, allowed for the generation of a single well-defined ICL in the duplex DNA. Examination of the resulting molecule confirmed complete crosslinking (Figure 1C). Using the crosslinked DNA, we then prepared two chromatography columns, one containing non-crosslinked DNA and the other containing the identical DNA sequence, now crosslinked. HeLa cells were treated with mitomycin C (MMC) to activate the ICL repair pathways, and nuclear extract was prepared. Using the two columns, we purified nuclear proteins interacting with regular DNA or ICL-containing DNA and analyzed them by SDS-PAGE followed by silver stain. As expected, we observed a number of polypeptides that interacted equally well with both DNA structures (Figure 1D). However, one polypeptide was more abundant in the sample from DNA containing an ICL than in the sample from the control DNA, migrating with an apparent molecular weight of 95 kDa (Figure 1D, lane 2). We excised the band corresponding to this polypeptide, and subsequent mass spectrometry (MS) analysis of the band resulted in 76 and 11 peptides from the UHRF1 and UHRF2 proteins, respectively (Figure 1E). UHRF1 (also known as RNF106 and NP95) is a RING E3 ubiquitin ligase.

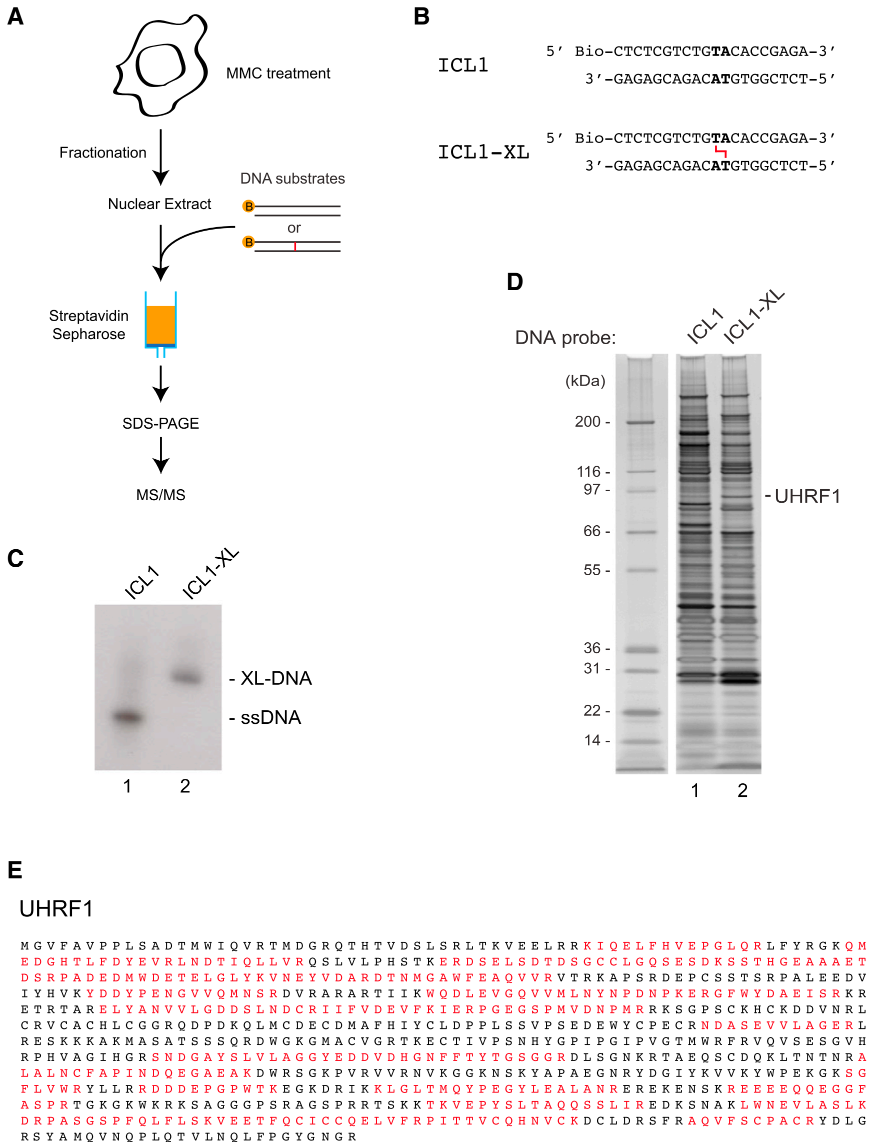

Figure 1. Purification of UHRF1

(A) Purification scheme for ICL-interacting proteins. Biotinylated DNA substrate is incubated with HeLa nuclear extract and captured by streptavidin-sepharose beads. The bound proteins are eluted, separated by SDS-PAGE, and analyzed by silver stain.

(B) Schematic of the biotinylated ICLs used in the purification.

(C) Analysis of the crosslinked ICL1 on an 8 M urea 20% polyacrylamide gel.

(D) Proteins purified from HeLa nuclear extract were stained by silver stain. The polypeptide identified by MS is indicated.

(E) List of identified peptides that match UHRF1. There were 76 peptides derived from UHRF1. Amino acids contained in the identified peptides are indicated in red.

UHRF2 is highly similar to UHRF1. Given the ~7-fold higher abundance of UHRF1 compared with UHRF2, we chose to focus further investigation on UHRF1. UHRF1 has been described as a protein that interacts directly with hemimethylated DNA, and has a higher affinity for hemimethylated than for unmodified or fully methylated DNA. As such, the protein was shown to recruit the DNA methyltransferase DNMT1 to newly replicated DNA, mediating methylation of the unmethylated, newly synthesized DNA strand, thereby ensuring the maintenance of methylated CpG sequences (Sharif et al., 2007).

UHRF1 Interacts Directly with DNA ICLs

Our data show an enrichment of UHRF1 bound to the ICL-containing DNA. Given that UHRF1 possesses DNA binding activity toward methylated DNA, we speculated that this protein might also recognize and directly interact with ICLs. However, we performed our purification using HeLa nuclear extract, raising the possibility that UHRF1 interacted indirectly with the ICL. In order to test whether UHRF1 interacts directly with the ICL, we set out to establish an in vitro DNA binding assay. We expressed full-length UHRF1 protein in Sf9 cells and purified it to homogeneity (Figure 2A). A biotinylated ICL-containing DNA molecule, ICL2 (Figure 2B), which is 60 bp long, was incubated with recombinant UHRF1 protein. Protein bound to DNA was purified using streptavidin-coated beads. UHRF1 was not bound to beads in the absence of DNA, and was bound weakly to non-crosslinked ICL2 DNA (Figure 2C, lanes 2 and 3). As expected, substantially more protein was bound to the ICL-containing DNA (Figure 2C, lane 4). Another E3 ligase, FANCL, did not display preferential binding, and thus served as a negative control for the experiment (Figure 2C, lanes 7 and 8). These data demonstrate a direct interaction between UHRF1 and the ICL.

To further reinforce these findings, we examined the ICL binding activity of UHRF1 by performing an electrophoretic mobility shift assay (EMSA). To be able to directly compare binding to ICL-containing DNA with binding to hemimethylated DNA, we designed two DNA molecules that are identical except for the central two base pairs. In one molecule, named ICL8, these central bases are TA; in the other, named CpG3, the bases are CG (Figure 2D). This allows for the introduction of either an ICL or methyl-C in the center of either molecule. As expected, UHRF1 bound weakly to the unmodified ICL8 probe (Figure 2E, lane 5). In contrast, the protein formed a strong complex with the ICL-containing ICL8 probe (Figure 2E, lane 6). Our experiments also confirmed that UHRF1 interacts better with hemimethylated DNA than with unmodified DNA (Figure 2E, lanes 7 and 8). We

OPEN ACCESS
CellPress

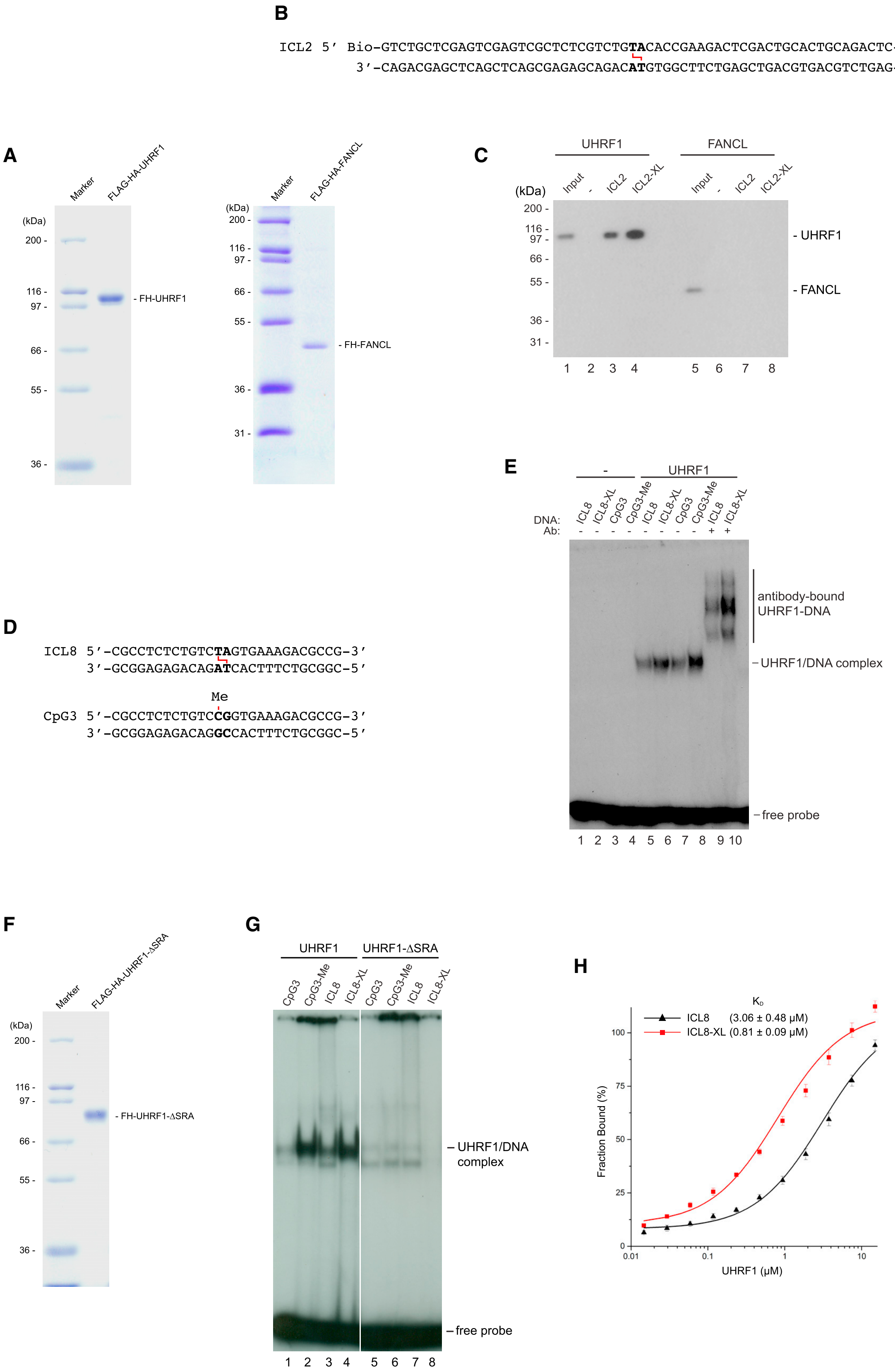

(legend on next page)

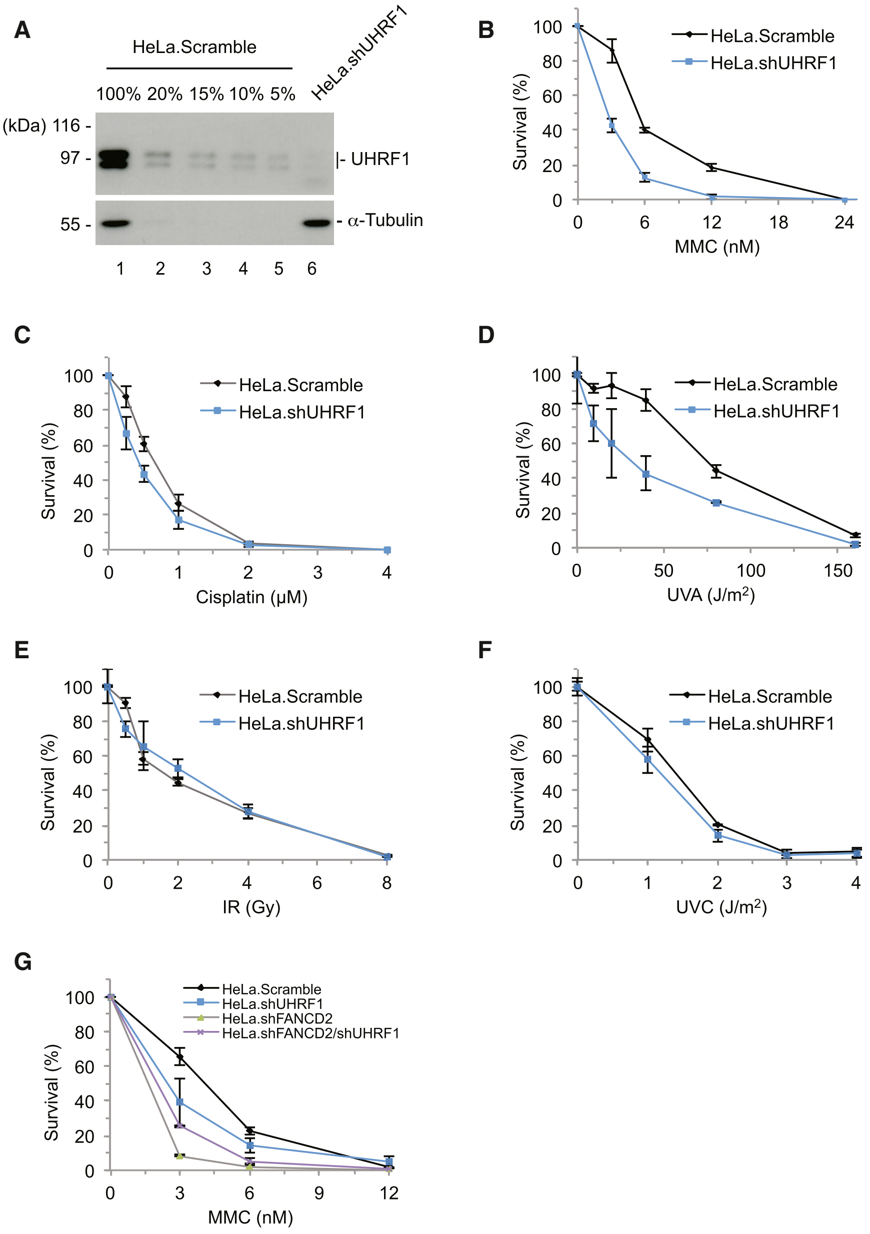

Figure 3. Knockdown of UHRF1 Sensitizes Cells to MMC

(A) A quantitative western blot analysis comparing the serial dilution of the lysate of HeLa cells expressing non-targeting shRNA (HeLa.Scramble), and HeLa cells expressing shRNA targeting UHRF1 (HeLa.shUHRF1), determines the efficiency of UHRF1 knockdown to be ~95%.

(B) A clonogenic survival assay of HeLa.Scramble and HeLa.shUHRF1 cells shows that UHRF1 is required for cell survival after MMC treatment.

(C) A clonogenic survival assay of HeLa.Scramble and HeLa.shUHRF1 cells shows that UHRF1 is partly required for cell survival after cisplatin treatment.

(D) A clonogenic survival assay of HeLa.Scramble and HeLa.shUHRF1 cells shows that UHRF1 is required for cell survival after TMP/UVA treatment.

(E) A clonogenic survival assay of HeLa.Scramble and HeLa.shUHRF1 cells shows that UHRF1 is not required for cell survival after IR treatment.

(F) A clonogenic survival assay of HeLa.Scramble and HeLa.shUHRF1 cells shows that UHRF1 is partly required for cell survival after UVC treatment.

(G) A clonogenic survival assay of HeLa.Scramble, HeLa.shFANCD2, HeLa.shUHRF1, and HeLa.shFANCD2/shUHRF1 shows that UHRF1 and FANCD2 are epistatic.

Error bars in (B)–(G) show SD. See also Figure S1.

confirmed the specificity of the protein-DNA complex using antibodies that specifically recognize the hemagglutinin (HA)-tag of the recombinant UHRF1 protein. Upon addition of these antibodies to the EMSA reaction, we observed a complete disappearance of the observed complex. Simultaneously, a slower-migrating complex appeared, demonstrating that the complex is composed of UHRF1 and DNA (Figure 2E, lanes 9 and 10).

The SRA domain of UHRF1 was previously shown to be required for the interaction with hemimethylated DNA (Arita et al., 2008). Thus, to gain further mechanistic insight into how UHRF1 recognizes ICLs, we introduced a deletion in the SRA domain and then assessed the ability of the resulting protein to interact with the ICL. Recombinant UHRF1-ΔSRA was purified (Figure 2F) and subjected to EMSA analysis. As expected, deleting the SRA domain abrogated the interaction with the ICL (Figure 2G).

Figure 2. UHRF1 Interacts Directly with DNA ICLs

(A) Coomassie blue stain of recombinant FLAG-HA-tagged UHRF1 and FLAG-HA-tagged FANCL purified from Sf9 cells.

(B) Schematic of the biotinylated ICL2 used in the in vitro DNA binding assay in (C).

(C) In vitro DNA binding assay showing that recombinant UHRF1 binds more strongly to crosslinked ICL2-XL DNA than to normal ICL2 DNA.

(D) Schematic of ICL8 and CPG3 DNA substrates used in (E).

(E) EMSA showing that UHRF1 forms stronger protein-DNA complexes with crosslinked ICL8-XL and hemimethylated CPG3-Me DNA substrates than with the corresponding unmodified DNA molecules. Super-shift using antibody against the HA-tag on recombinant UHRF1 confirms that the protein/DNA complex is formed by UHRF1.

(F) Coomassie blue stain of recombinant FLAG-HA-tagged UHRF1-ΔSRA purified from Sf9 cells.

(G) EMSA using recombinant UHRF1-ΔSRA lacking the SRA domain shows that the SRA domain of UHRF1 is responsible for the interaction with the ICL.

(H) Fluorescence anisotropy assay determining the characteristics of UHRF1 binding to either ICL8 or ICL8-XL. Normalized and averaged anisotropy ± SEM.

OPEN
ACCESS
CellPress

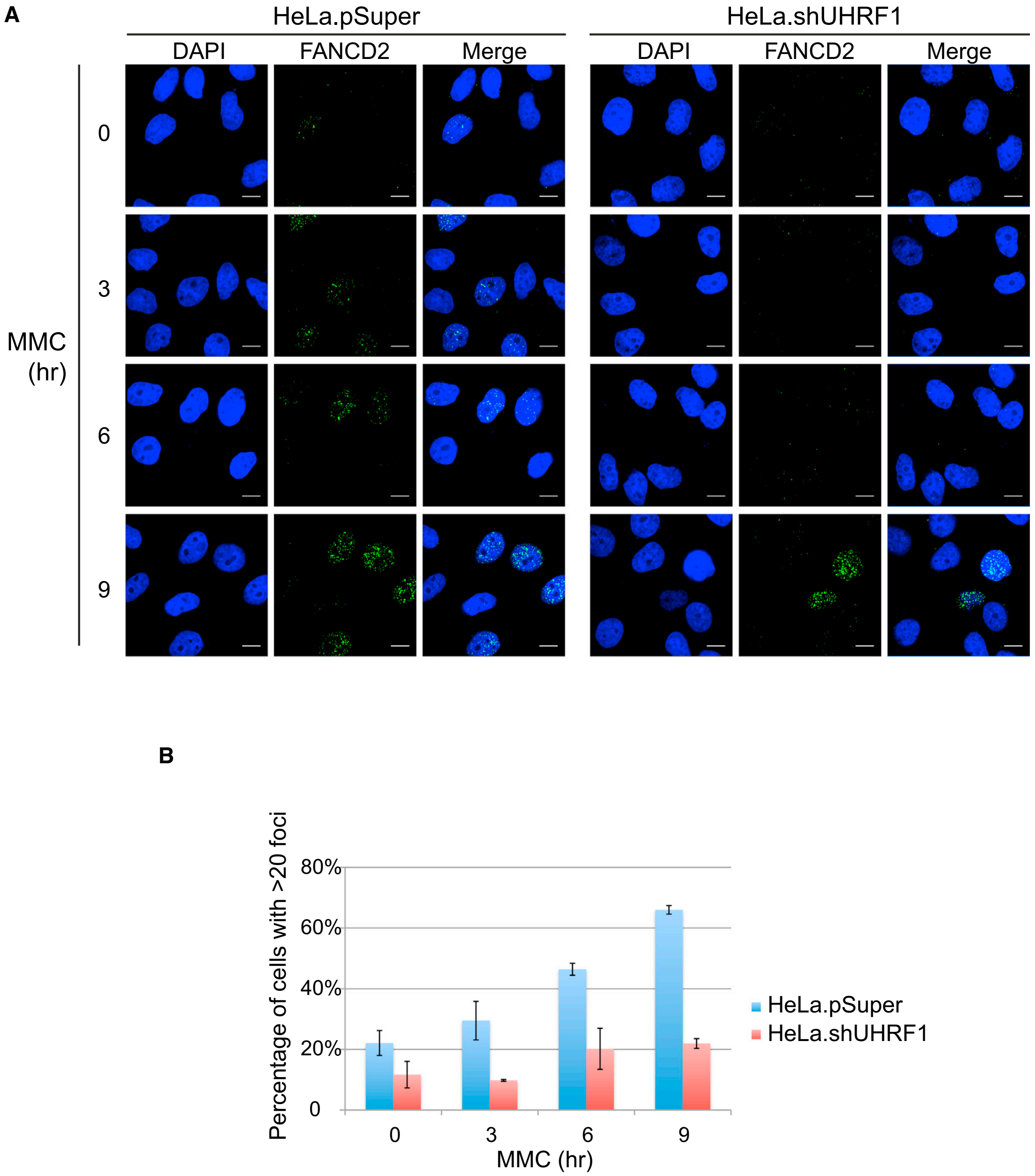

Figure 4. UHRF1 Is Required for Normal FANCD2 Foci Formation In Vivo

(A) FANCD2 foci accumulate after MMC treatment in control HeLa cells, whereas FANCD2 foci formation is defective in the absence of UHRF1. Scale bar, 10 μm.

(B) Quantification of the percentage of cells with more than 20 foci per cell. The error bars are calculated based on two individual experiments and show SD. See also Figure S1.

These experiments, which were conducted using two separate methods, reveal that UHRF1 has a stronger interaction with ICL-containing DNA than with normal DNA. However, to gain additional information about the relative affinities, we applied fluorescence anisotropy to determine the respective K_D values. The DNA molecules were 5′ labeled with Alexa Fluor 488, and the interaction of UHRF1 with DNA was monitored as protein concentrations were increased. We determined the K_D of the interaction between UHRF1 and the ICL-containing DNA as 0.81 μM. The corresponding K_D value for the interaction with normal DNA was significantly higher (3.06 μM; Figure 2H), demonstrating that UHRF1 has a stronger affinity for ICL-containing DNA than for undamaged DNA.

Taken together, these data show that UHRF1 interacts directly with an ICL in vitro via its SRA domain.

OPEN ACCESS
CellPress

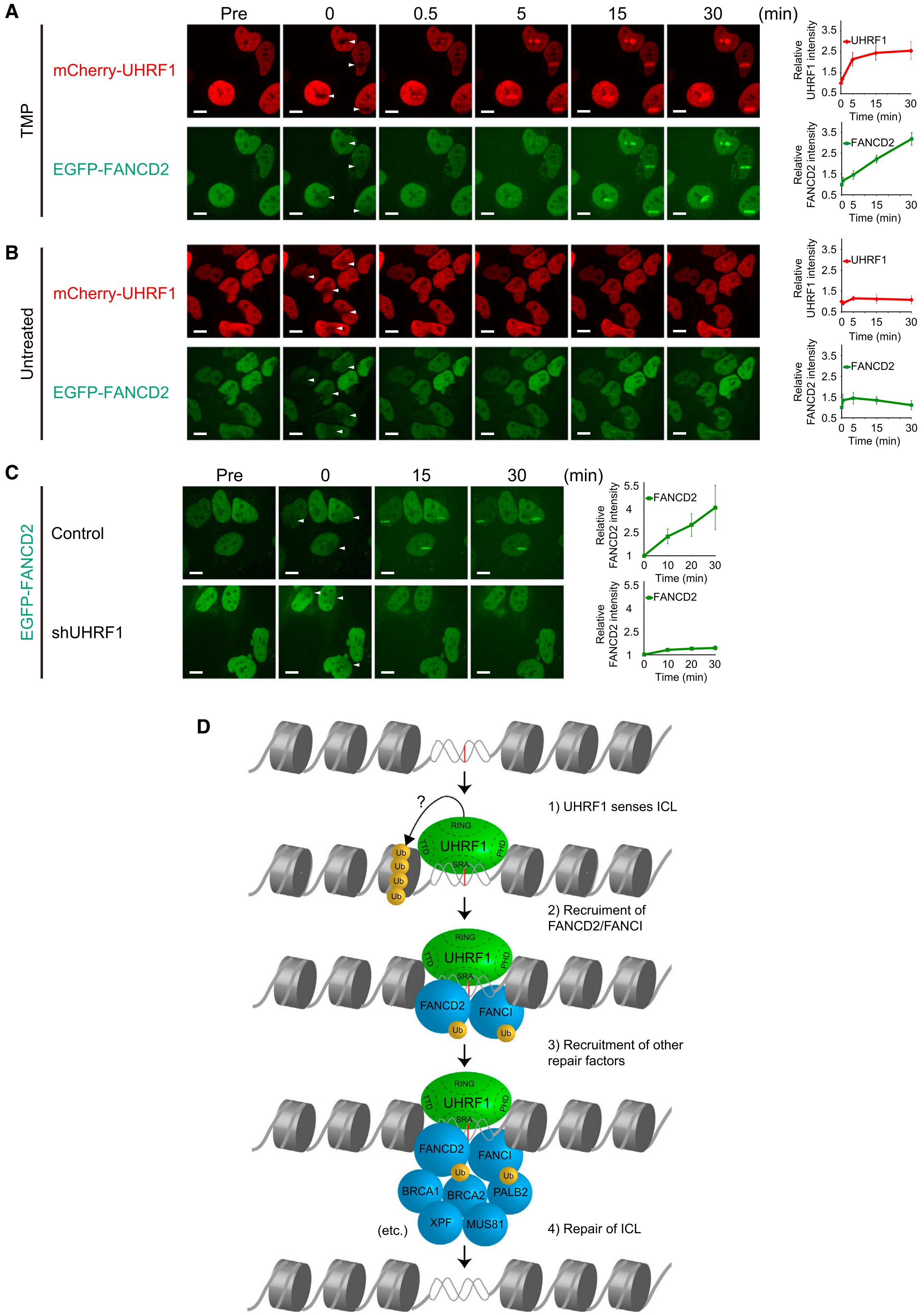

(legend on next page)

Knockdown of UHRF1 Sensitizes Cells to MMC

Since UHRF1 is able to recognize and bind directly to ICLs in vitro, we speculated that the protein might be involved in the cellular response to ICLs in vivo. The FA DNA repair pathway responds to ICLs, involving 17 FA proteins in cooperation with a number of other non-FA DNA repair proteins. Therefore, knockdown of UHRF1 is expected to sensitize cells to MMC. We established a HeLa cell line in which the cellular level of UHRF1 is reduced to <5% using small hairpin RNA (shRNA) (Figure 3A). We then performed clonogenic survival assays and found that indeed knockdown of UHRF1 led to a significant decrease in survival in response to MMC (Figure 3B). We repeated this experiment in HEK293T cells and observed the same effect (data not shown). Surprisingly, UHRF1 knockdown cells were only mildly sensitive to cisplatin (Figure 3C). Given the difference in sensitivity to MMC and cisplatin, we sought to induce ICLs and as little as possible of other types of DNA damage (e.g., mono-adducts and single- or double-strand breaks). One of the best ways to achieve this is to use TMP in combination with UVA (Huang et al., 2013). As expected, we observed a clear sensitivity to this more homogeneous ICL DNA damage (Figure 3D). We also assessed the cellular sensitivity to other types of DNA damage, and found only slight sensitivity to IR and UVC (Figures 3E and 3F). In a previous study, Muto et al. (2002) also described sensitivity of UHRF1-deficient cells to genotoxic agents, although they observed a greater sensitivity to IR and UVC. The increased sensitivity might be due to their use of mouse embryonic stem cells, rather than human cell lines, as were used in the present study.

Given the specific sensitivity of the UHRF1 knockdown cells to ICL-inducing agents, we suspected a possible functional connection to the FA DNA repair pathway. To test this directly, we investigated the cellular sensitivities following knockdown of UHRF1, FANCD2, or both. We found that cells depleted of FANCD2 (Figure S1A) were more sensitive to MMC than were cells depleted of UHRF1 (Figure 3G). However, depletion of UHRF1 in cells in which FANCD2 was already depleted did not further sensitize the cells. On the contrary, we found a slight suppression of sensitivity in these cells.

UHRF1 Is Required for Normal FANCD2 Foci Formation In Vivo

Given the epistatic relationship between UHRF1 and FANCD2, we speculated that FANCD2 nuclear foci formation could be dependent on UHRF1. To test this hypothesis, we treated control and knockdown cells with MMC and stained them for FANCD2 by immunofluorescence at various time points. The cell-cycle profile was comparable between the two cell lines (Figure S1B). Some control cells with increased numbers of foci were already visible after 3 hr, and the number of positive cells increased steadily for up to 9 hr (Figure 4A). In contrast, the knockdown cells displayed nearly no increase in foci formation at the 3-hr and 6-hr time points, and only a modest increase at the last time point at 9 hr. Quantification of the data showed that the percentage of cells with more than 20 foci rose to ~60% in control cells at 9 hr, whereas the corresponding number was only ~20% in knockdown cells (Figure 4B).

UHRF1 Is Rapidly Recruited to ICLs In Vivo and Precedes the Recruitment of FANCD2

Given that UHRF1 interacts directly with ICLs in vitro and is required for proper foci formation of FANCD2 in vivo, we speculated that UHRF1 itself is recruited to crosslinked DNA in vivo, and that this triggers the chromatin recruitment of FANCD2. To test this hypothesis directly, we turned to live-cell imaging using fluorophore-tagged proteins. UHRF1 and FANCD2 were stably expressed in HeLa cells as fusion proteins with mCherry and EGFP, respectively. We introduced ICLs with a localized laser stripe after incubating the cells with TMP (Thazhathveetil et al., 2007). We observed that UHRF1 was recruited to ICLs very quickly and formed a clear stripe within 30 s. FANCD2 was also recruited, albeit slightly more slowly than UHRF1, and formed a visible stripe within 5 min (Figure 5A). Importantly, there was no recruitment of either one of the proteins in the absence of TMP (Figure 5B). These data encouraged us to directly test, in live cells, whether UHRF1 mediates the recruitment of FANCD2 to ICLs. Using control and UHRF1 knockdown cells, we assessed the recruitment of FANCD2 to ICLs in the presence and absence of UHRF1. Strikingly, we found that knockdown of UHRF1 completely abolished FANCD2 recruitment (Figure 5C).

These experiments further reinforce the notion that UHRF1 is recruited directly to ICLs, and that this recruitment is required for the subsequent recruitment of FANCD2.

DISCUSSION

Repair of an ICL requires the recruitment of FANCD2 to the site of damage (Hu et al., 2011; Knipscheer et al., 2009). The mechanism of this critical recruitment is unknown. Here, we show that UHRF1 is recruited very quickly to ICLs in vivo, and that the recruitment both precedes and is required for the recruitment of FANCD2 to the ICL.

UHRF1 Is a Sensor for ICLs

UHRF1 interacts directly with the ICL through an SRA nucleic acid interaction domain. The SRA domain was previously reported to be necessary for the interaction with hemimethylated DNA (Arita et al., 2008), facilitating the recruitment of DNMT1 to newly replicated DNA (Bostick et al., 2007). Using in vitro

Figure 5. UHRF1 Is Rapidly Recruited to ICLs In Vivo and Precedes the Recruitment of FANCD2

(A and B) HeLa cells expressing mCherry-tagged UHRF1 and EGFP-tagged FANCD2 were (A) pre-treated with TMP or (B) untreated, and microirradiated at the indicated areas (white arrows). Charts on the right show quantification of mCherry-UHRF1 and EGFP-FANCD2 at the ICL sites. UHRF1 and FANCD2 were recruited to TMP-induced ICLs sites (A), but not to irradiated sites in the absence of TMP (B). Scale bar, 10 μm.

(C) HeLa cells expressing EGFP-tagged FANCD2 with or without UHRF1 knockdown were microirradiated at the indicated areas (white arrows). Depletion of UHRF1 abrogates the rapid accumulation of FANCD2 at the ICLs. Scale bar, 10 μm. Charts on the right show quantification of EGFP-FANCD2 at the ICL sites.

(D) Model showing how UHRF1 is recruited to the ICL, facilitating the recruitment of FANCD2, which again precedes the recruitment of additional DNA repair factors. See also Figure S1.

protein-DNA binding assays, we found that UHRF1 interacts roughly as strongly with hemimethylated DNA as with a DNA probe containing a single ICL. In vivo, when the DNA is in the context of chromatin, UHRF1 forms additional contacts with histone H3 through its TTD and PHD domains (Gelato et al., 2014; Xie et al., 2012), which facilitate its recruitment to chromatin. The combination of these three interactions is likely to ensure both stronger binding and higher specificity.

Specificity of UHRF1 for Different Types of ICLs

Interestingly, our studies uncovered that UHRF1 responds to ICLs formed by the psoralen compound TMP as well as to ICLs formed by MMC, but less so to ICLs formed by cisplatin. ICLs formed by either TMP or MMC cause minor distortions of the DNA helix, whereas ICLs formed by cisplatin cause a major distortion (Guainazzi and Schärer, 2010). Thus, it is possible that UHRF1 specifically recognizes ICLs with minor distortions. Structural studies of the UHRF1/ICL complex will help to elucidate the atomic nature of this protein-DNA interaction. Both psoralens and MMC are found in nature, whereas cisplatin is a chemically synthesized compound not found in nature. The observed specificity might reflect evolution of the FA pathway toward its current form of ICL repair in humans.

UHRF1 Is Recruited to ICLs In Vivo and Is Required for the Recruitment of FANCD2

We found that UHRF1 is recruited to ICLs in vivo within seconds of their appearance in the chromosomes. We also found that this recruitment precedes and is required for the recruitment of FANCD2. It is likely that UHRF1 is recruited directly to the ICL, thereby forming a landing platform for FANCD2 (Figure 5D). FANCD2 also possesses DNA binding activity itself (Joo et al., 2011), and it might be that the combination of this basal DNA binding and a potential interaction with UHRF1 allows for a stable retention of FANCD2 at the ICL. Repair of ICLs requires the recruitment of FANCD2, which allows for the recruitment of XPF/ERCC1 and other nucleases (Hu et al., 2011). Therefore, it is possible that the recruitment of UHRF1 and subsequently FANCD2 precedes the recruitment of specific endonucleases required for ICL repair, in good agreement with recent reports (Hodskinson et al., 2014; Klein Douwel et al., 2014). The small gap in time between the recruitment of UHRF1 and FANCD2 might also allow for the recruitment of other factors; for instance, it was shown that NER factors are recruited to an ICL preceding the recruitment of some FA proteins (Shen et al., 2009).

Conclusions

In conclusion, we present a mechanism for sensing DNA ICLs. We show that UHRF1 specifically recognizes and binds to ICLs within seconds after their appearance in the genome, and that this recruitment allows for the subsequent recruitment of FANCD2, permitting the DNA repair process to initiate.

EXPERIMENTAL PROCEDURES

Cell Lines, Antibodies, and Plasmids

HeLa and HEK293T cells were grown in DMEM (D5796; Sigma-Aldirch) supplemented with 2.5%–10% fetal bovine serum (FBS). The following antibodies were used: anti-UHRF1 (sc-373; Santa Cruz Biotechnology), anti-FANCD2 (sc-20022; Santa Cruz Biotechnology), anti-α-tubulin (5829; Millipore), and anti-HA (mouse monoclonal antibody clone 12CA5).

EGFP-fused FANCD2 and mCherry-fused UHRF1 cDNA were expressed using the pOZ-N plasmid (Nakatani and Ogryzko, 2003). shRNA-mediated knockdown of the UHRF1 and FANCD2 genes was achieved by expressing the target sequence 5′-AGATATAACGTTAGGGTTT-3′ and 5′-GAGCAAAGC CACTGAGGTA-3′, respectively, in the pSuper.retro vector (Clontech). Transfections of plasmid DNA were carried out using FuGENE6 (Promega) according to the manufacturer's instructions. The UHRF1 SRA domain deletion plasmid was generated as above, with amino acids 427–630 deleted.

Preparation of Interstrand Crosslinked DNA Substrates

The DNA oligos were annealed in a buffer containing 10 mM Tris-HCl (pH 7.5), 100 mM NaCl, and 1 mM EDTA. TMP (T6137, Sigma-Aldrich)/UVA (365 nm) crosslinking induction was performed as described previously (Esposito et al., 1988). Interstrand crosslinking was confirmed by 8M urea 20% denaturing PAGE.

ICL-Binding Protein Purification and UHRF1-DNA In Vitro Binding

Nuclear proteins were extracted as previously described (Dignam et al., 1983). Then, 1 mg of nuclear extract from HeLa cells or 3 μg of recombinant UHRF1/FANCL from Sf9 cells was incubated with 25 pmol biotin-labeled DNA substrates (ICL1 and ICL1-XL were used for purification from nuclear extract, and ICL2 and ICL2-XL were used for UHRF1/FANCL in vitro binding experiments). The binding buffer contained 5 mM Tris (pH 7.9), 30 mM KCl, 1 mM DTT, 10 mM HEPES-KOH (pH 7.9), 1 mM EDTA, 5% glycerol, and 0.3 mg/ml BSA (New England BioLabs). The protein and DNA probe mix was incubated at 30°C for 15 min and then mixed with 10 μl of 50% streptavidin sepharose (GE Healthcare). The matrix with streptavidin beads was washed with several column volumes before elution. Eluted proteins were analyzed by electrophoresis on a 4%–12% NuPage Bis-Tris gradient gel (Life Technologies) and visualized by silver stain (Silver Quest; Life Technologies). For UHRF1 and FANCL in vitro DNA binding, the proteins were analyzed by electrophoresis on 10% SDS-PAGE gel followed by western blot analysis.

MS Analysis

Proteins were reduced with DTT, cysteine residues were derivatized with iodoacetamide, and the proteins were separated by SDS-PAGE. Proteins from silver-stained gel bands were in-gel digested with trypsin (Shevchenko et al., 1996). The generated peptide mixtures were subjected to liquid chromatography-tandem MS (LC-MS/MS) using a hybrid linear ion trap/FT-ICR mass spectrometer (LTQ FT; Thermo Electron) essentially as described previously (Haas et al., 2006). MS/MS spectra were assigned by searching them with the SEQUEST algorithm (Eng et al., 1994) against the human International Protein Index sequence database.

Protein Purification

Proteins purified from Sf9 cells were expressed using the pFastBac1 vector (Life Technologies) with an engineered N-terminal Flag-HA tag. Cell pellets were resuspended in lysis buffer (20 mM Tris-HCl (pH 8.0), 0.1 M KCl, 10% glycerol, 0.1% Tween-20, 2 mM β-ME, and 0.2 mM phenylmethanesulfonyl fluoride). Lysates were clarified by centrifugation and the supernatants were incubated with M2 anti-FLAG agarose resin for 2 hr. The resin was washed extensively and the protein was eluted in the same buffer containing 0.5 mg/ml FLAG peptide, but excluding Tween-20.

EMSA

EMSA was performed as previously described (Cohn et al., 2001) with the following modifications: the binding reaction that contained 1 μg of UHRF1 and 1 nM of radiolabeled DNA was performed in 10 μl of a solution containing 14 mM Tris-HCl (pH 8.0), 100 mM NaCl, 3.4% glycerol, 1 mM DTT, 20 ng poly(dI·dC)-poly(dI·dC), and 1 μg BSA (New England BioLabs). For supershift, 2 μg anti-HA antibody was added.

Fluorescence Anisotropy

Recombinant Flag-tagged UHRF1 was incubated with 10 nM ICL8 or ICL-XL (Eurofins Genomics) labeled with Alexa Fluor 488 on the 5′ terminus in a buffer

containing 20 mM Tris-HCl (pH 8.0), 100 mM KCl, 1 mM DTT, and 0.2 mg/ml BSA on ice for 1 hr. The reaction volume was 40 μl. A PHERAstar FS fluorimeter (BMG Labtech; λex = 490 nm, λem = 520 nm) was used to record the fluorescence polarization. Data were fitted with Origin software (OriginLab) with a one-site specific binding equation.

Clonogenic Survival Assay

Cells (250–4,000) were plated in six-well plates and treated with different dosages of the indicated damaging agents on the next day. For TMP/UVA treatment, the cells were treated with 50 ng/ml TMP for 30 min and irradiated with the indicated UVA dosages. Colony formation was scored after 10–14 days using 1% (w/v) crystal violet in methanol.

Preparation of Whole-Cell Lysate

Cells were scraped off the dishes and centrifuged at 1,000 rpm for 5 min. Cell pellets were resuspended and incubated in an equal volume of Benzonase buffer (2 mM $MgCl_2$, 20 mM Tris-HCl [pH 8.0], 10% glycerol, 1% Triton X-100, and 12.5 U/ml Benzonase; (E1014, Sigma-Aldrich) on ice for 10 min. The cells were then lysed by addition of an equal volume of 2% SDS to reach a final concentration of 1%. Samples were heated at 70°C for 2 min. The protein concentration was determined by Bradford assay (Bio-Rad Life Science).

Immunofluorescence Microscopy

HeLa cells were grown on coverslips, pre-extracted with cold PBS/1% Triton X-100 on ice for 10 min, and fixed with 4% (w/v) paraformaldehyde for 10 min at 25°C. FANCD2 foci were detected using an antibody against FANCD2 (FI-17; 1:100) in 5% (w/v) BSA in PBS, and visualized using Alexa Fluor 488-conjugated secondary antibody (A21202; 1:1,000; Life Technologies). The cells were fixed again using 4% paraformaldehyde after staining, and mounted with DAPI-containing mounting medium (Vector Laboratories). Imaging was carried out using the DeltaVision System (Applied Precision) installed with Resolve3D SoftWoRx-Acquire Version 4.0.0. A 60× optic objective was selected (Olympus 60X/1.42, PlanApo, N). Fluorescent images were captured with a camera (CoolSNAP HQ/ICX285).

Live-Cell Imaging

EGFP-fused FANCD2 and mCherry-fused UHRF1 cDNA were inserted into the pOZ vector as described above. Live-cell imaging was carried out with an Olympus IX81 microscope connected to a PerkinElmer UltraView Vox spinning-disk system equipped with a Plan-Apochromat 60×/1.4 oil objective using Volocity software 6.3 for image capture. EGFP and mCherry were excited with 488 nm and 561 nm laser lines, respectively. Throughout the experiment, the cells were maintained at 5% CO_2 and 37°C using a live-cell environmental chamber (Tokai hit). Confocal image series typically were recorded with a frame size of 512 × 512 pixels and a pixel size of 139 nm. For localized DNA damage induction, cells were seeded in a glass-bottom dish (MatTek) and sensitized by incubation in DMEM supplemented with 2.5% FBS and 500 ng/ml TMP for 30 min at 37°C. Microirradiation was performed using the FRAP preview mode of the Volocity software by scanning three to five preselected stripes (50 × 3 pixels, 100 ms for each irradiation time) within the nucleus 80 times with a 405-nm laser set to 100% laser power. The mCherry and EGFP intensities at microirradiated sites were quantified using ImageJ with Fiji and normalized by their intensities before microirradiation.

SUPPLEMENTAL INFORMATION

Supplemental Information includes one figure and can be found with this article online at http://dx.doi.org/10.1016/j.celrep.2015.02.053.

AUTHOR CONTRIBUTIONS

C.-C.L., B.Z., Y.Y., W.H., S.P.G., and M.A.C. designed and analyzed the experiments. C.-C.L., B.Z., Y.Y., and W.H. performed the experiments. C.-C.L., B.Z., Y.Y., and M.A.C. prepared the manuscript.

ACKNOWLEDGMENTS

The authors thank members of the M.A.C. laboratory, Johannes Walter, Gianluca Veggiani, Christopher Schoene, and Deepa Nath for reading and discussing the manuscript. We also thank the staff at the Micron Microscopy Facility for excellent help and support, and George Hatzopoulos and David Staunton for help with fluorescence anisotropy. This work was supported by grants UF100717 and ALRXNV0 from the Royal Society (M.A.C.), grant 103/789 from The Fell Fund (M.A.C.), grant 090087/Z/09/Z from The Wellcome Trust (B.Z. and M.A.C.), grant OCRC0213-MC from the OCRC/CR-UK (M.A.C.), the Taiwanese Government (C.-C.L.), a Goodger scholarship (C.-C.L.), and JSPS (Y.Y.).

Received: July 10, 2014
Revised: February 12, 2015
Accepted: February 23, 2015
Published: March 19, 2015

REFERENCES

Arita, K., Ariyoshi, M., Tochio, H., Nakamura, Y., and Shirakawa, M. (2008). Recognition of hemi-methylated DNA by the SRA protein UHRF1 by a base-flipping mechanism. Nature *455*, 818–821.

Bostick, M., Kim, J.K., Estève, P.O., Clark, A., Pradhan, S., and Jacobsen, S.E. (2007). UHRF1 plays a role in maintaining DNA methylation in mammalian cells. Science *317*, 1760–1764.

Ciccia, A., Ling, C., Coulthard, R., Yan, Z., Xue, Y., Meetei, A.R., Laghmani, H., Joenje, H., McDonald, N., de Winter, J.P., et al. (2007). Identification of FAAP24, a Fanconi anemia core complex protein that interacts with FANCM. Mol. Cell *25*, 331–343.

Cohn, M.A., and D'Andrea, A.D. (2008). Chromatin recruitment of DNA repair proteins: lessons from the fanconi anemia and double-strand break repair pathways. Mol. Cell *32*, 306–312.

Cohn, M.A., Hjelmsø, I., Wu, L.C., Guldberg, P., Lukanidin, E.M., and Tulchinsky, E.M. (2001). Characterization of Sp1, AP-1, CBF and KRC binding sites and minisatellite DNA as functional elements of the metastasis-associated mts1/S100A4 gene intronic enhancer. Nucleic Acids Res. *29*, 3335–3346.

Dignam, J.D., Martin, P.L., Shastry, B.S., and Roeder, R.G. (1983). Eukaryotic gene transcription with purified components. In Methods in Enzymology, R. Wu, L. Grossman, and K. Moldave, eds. (Academic Press), pp. 582–598.

Eng, J.K., McCormack, A.L., and Yates, J.R. (1994). An approach to correlate tandem mass spectral data of peptides with amino acid sequences in a protein database. J. Am. Soc. Mass Spectrom. *5*, 976–989.

Esposito, F., Brankamp, R.G., and Sinden, R.R. (1988). DNA sequence specificity of 4,5′,8-trimethylpsoralen cross-linking. Effect of neighboring bases on cross-linking the 5′-TA dinucleotide. J. Biol. Chem. *263*, 11466–11472.

Gelato, K.A., Tauber, M., Ong, M.S., Winter, S., Hiragami-Hamada, K., Sindlinger, J., Lemak, A., Bultsma, Y., Houliston, S., Schwarzer, D., et al. (2014). Accessibility of different histone H3-binding domains of UHRF1 is allosterically regulated by phosphatidylinositol 5-phosphate. Mol. Cell *54*, 905–919.

Guainazzi, A., and Schärer, O.D. (2010). Using synthetic DNA interstrand crosslinks to elucidate repair pathways and identify new therapeutic targets for cancer chemotherapy. Cell. Mol. Life Sci. *67*, 3683–3697.

Haas, W., Faherty, B.K., Gerber, S.A., Elias, J.E., Beausoleil, S.A., Bakalarski, C.E., Li, X., Villén, J., and Gygi, S.P. (2006). Optimization and use of peptide mass measurement accuracy in shotgun proteomics. Mol. Cell. Proteomics *5*, 1326–1337.

Hodskinson, M.R., Silhan, J., Crossan, G.P., Garaycoechea, J.I., Mukherjee, S., Johnson, C.M., Schärer, O.D., and Patel, K.J. (2014). Mouse SLX4 is a tumor suppressor that stimulates the activity of the nuclease XPF-ERCC1 in DNA crosslink repair. Mol. Cell *54*, 472–484.

Hu, L., Li, Z., Wang, P., Lin, Y., and Xu, Y. (2011). Crystal structure of PHD domain of UHRF1 and insights into recognition of unmodified histone H3 arginine residue 2. Cell Res. *21*, 1374–1378.

Huang, J., Liu, S., Bellani, M.A., Thazhathveetil, A.K., Ling, C., de Winter, J.P., Wang, Y., Wang, W., and Seidman, M.M. (2013). The DNA translocase FANCM/MHF promotes replication traverse of DNA interstrand crosslinks. Mol. Cell *52*, 434–446.

Joo, W., Xu, G., Persky, N.S., Smogorzewska, A., Rudge, D.G., Buzovetsky, O., Elledge, S.J., and Pavletich, N.P. (2011). Structure of the FANCI-FANCD2 complex: insights into the Fanconi anemia DNA repair pathway. Science *333*, 312–316.

Klein Douwel, D., Boonen, R.A., Long, D.T., Szypowska, A.A., Räschle, M., Walter, J.C., and Knipscheer, P. (2014). XPF-ERCC1 acts in Unhooking DNA interstrand crosslinks in cooperation with FANCD2 and FANCP/SLX4. Mol. Cell *54*, 460–471.

Knipscheer, P., Räschle, M., Smogorzewska, A., Enoiu, M., Ho, T.V., Schärer, O.D., Elledge, S.J., and Walter, J.C. (2009). The Fanconi anemia pathway promotes replication-dependent DNA interstrand cross-link repair. Science *326*, 1698–1701.

Muniandy, P.A., Thapa, D., Thazhathveetil, A.K., Liu, S.T., and Seidman, M.M. (2009). Repair of laser-localized DNA interstrand cross-links in G1 phase mammalian cells. J. Biol. Chem. *284*, 27908–27917.

Muto, M., Kanari, Y., Kubo, E., Takabe, T., Kurihara, T., Fujimori, A., and Tatsumi, K. (2002). Targeted disruption of Np95 gene renders murine embryonic stem cells hypersensitive to DNA damaging agents and DNA replication blocks. J. Biol. Chem. *277*, 34549–34555.

Nakatani, Y., and Ogryzko, V. (2003). Immunoaffinity purification of mammalian protein complexes. Methods Enzymol. *370*, 430–444.

Räschle, M., Knipscheer, P., Enoiu, M., Angelov, T., Sun, J., Griffith, J.D., Ellenberger, T.E., Schärer, O.D., and Walter, J.C. (2008). Mechanism of replication-coupled DNA interstrand crosslink repair. Cell *134*, 969–980.

Sharif, J., Muto, M., Takebayashi, S., Suetake, I., Iwamatsu, A., Endo, T.A., Shinga, J., Mizutani-Koseki, Y., Toyoda, T., Okamura, K., et al. (2007). The SRA protein Np95 mediates epigenetic inheritance by recruiting Dnmt1 to methylated DNA. Nature *450*, 908–912.

Shen, X., Do, H., Li, Y., Chung, W.H., Tomasz, M., de Winter, J.P., Xia, B., Elledge, S.J., Wang, W., and Li, L. (2009). Recruitment of fanconi anemia and breast cancer proteins to DNA damage sites is differentially governed by replication. Mol. Cell *35*, 716–723.

Shevchenko, A., Wilm, M., Vorm, O., and Mann, M. (1996). Mass spectrometric sequencing of proteins silver-stained polyacrylamide gels. Anal. Chem. *68*, 850–858.

Thazhathveetil, A.K., Liu, S.T., Indig, F.E., and Seidman, M.M. (2007). Psoralen conjugates for visualization of genomic interstrand cross-links localized by laser photoactivation. Bioconjug. Chem. *18*, 431–437.

Vasquez, K.M. (2010). Targeting and processing of site-specific DNA interstrand crosslinks. Environ. Mol. Mutagen. *51*, 527–539.

Xie, S., Jakoncic, J., and Qian, C. (2012). UHRF1 double tudor domain and the adjacent PHD finger act together to recognize K9me3-containing histone H3 tail. J. Mol. Biol. *415*, 318–328.

Cell Reports

Supplemental Information

UHRF1 Is a Sensor for DNA Interstrand Crosslinks and Recruits FANCD2 to Initiate the Fanconi Anemia Pathway

Chih-Chao Liang, Bao Zhan, Yasunaga Yoshikawa, Wilhelm Haas, Steven P. Gygi, and Martin A. Cohn

Figure S1

A

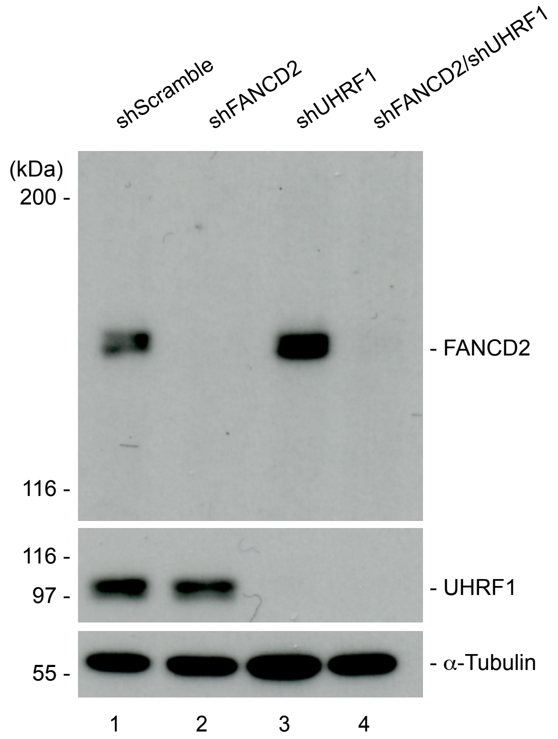
shScramble
shFANCD2
shUHRF1
shFANCD2/shUHRF1
(kDa)
200 -
116 -
116 -
97 -
55 -
- FANCD2
- UHRF1
- α-Tubulin
1
2
3
4

B

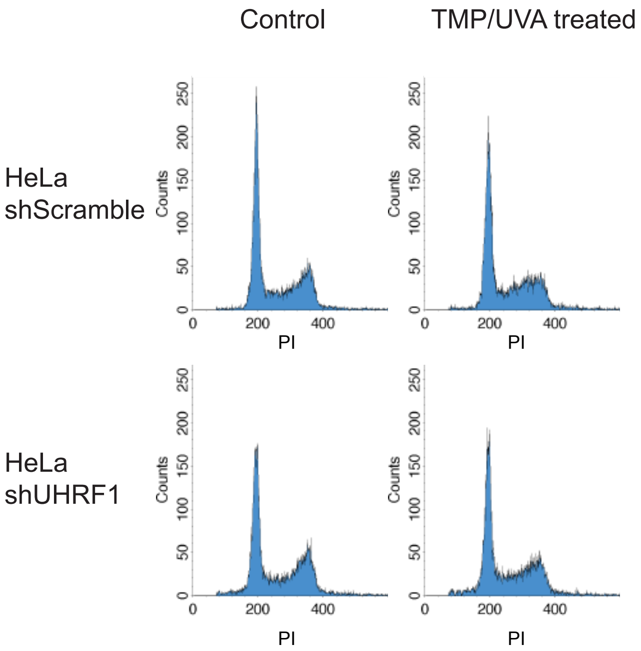
Control
TMP/UVA treated
HeLa shScramble
HeLa shUHRF1
Counts
PI
0
50
100
150
200
250
0
200
400

Supplementary figure legend

Figure S1, related to Figures 3, 4 and 5

A) Western blot analysis confirms the knock down level of UHRF1 and FANCD2 in the single and double knock down cells used in Figure 3G. B) HeLa.shScramble and HeLa.shUHRF1 were untreated or treated with TMP/UVA. Cells were harvested after 3 hours, stained with propidium iodide, and analyzed by FACS analysis.
